# Two hundred days of COVID-19 in São Paulo State, Brazil

**DOI:** 10.1017/S0950268820002927

**Published:** 2020-12-02

**Authors:** Gabriel Berg de Almeida, Micheli Pronunciate, Rejane Maria Tommasini Grotto, Edmur Azevedo Pugliesi, Raul Borges Guimarães, Thomas Nogueira Vilches, Renato Mendes Coutinho, Rafael de Castro Catão, Claudia Pio Ferreira, Carlos Magno Castelo Branco Fortaleza

**Affiliations:** 1 Clinical Hospital of Botucatu Medical School (HCFMB), Botucatu, Brazil; 2 Department of Infectious Diseases, São Paulo State University (UNESP), Botucatu Medical School, Botucatu, Brazil; 3 São Paulo State University (UNESP), School of Agriculture, Botucatu, Brazil; 4 São Paulo State University (UNESP), School of Technology and Sciences, Presidente Prudente, Brazil; 5 University of Campinas (UNICAMP), Institute of Mathematics, Statistics and Scientific Computation, Campinas, Brazil; 6 Federal University of ABC (UFABC), Center for Mathematics, Computation and Cognition, Santo André, Brazil; 7 Department of Geography, Federal University of Espírito Santo (UFES), Vitória, Brazil; 8 São Paulo State University (UNESP), Institute of Biosciences, Botucatu, Brazil

**Keywords:** COVID-19, emerging infections, epidemics, epidemiology, infectious disease epidemiology

## Abstract

Two hundred days after the first confirmed case of COVID-19 in Brazil, the epidemic has rapidly spread in metropolitan areas and advanced throughout the countryside. We followed the temporal epidemic pattern at São Paulo State, the most populous of the country, the first to have a confirmed case of COVID-19, and the one with the most significant number of cases until now. We analysed the number of new cases per day in each regional health department and calculated the effective reproduction number (*R_t_*) over time. Social distance measures, along with improvement in testing and isolating positive cases, general population mask-wearing and standard health security protocols for essential and non-essential activities, were adopted and impacted on slowing down epidemic velocity but were insufficient to stop transmission.

## Background and epidemiology

Severe acute respiratory syndrome coronavirus 2 (SARS-CoV-2) was first identified in Brazil on 25 February 2020, in São Paulo [1]. The most populous city of Latin America was the route for COVID-19 importation, mainly from the USA and Italy [2]. It took 17 days for Brazil to reach a hundred cases, mainly reported in the capitals highly connected by airports and with an intensive flux of people. Two hundred days after the first confirmed case, the epidemic rapidly spreads across the country, and the disease advances through the interior.

São Paulo implemented state-wide quarantine measures quite early in the epidemic course. On 24 March, the government adopted a social distance recommendation for all people associated with closing trade and non-essential services. The decree suggested that people's movement should be limited to the immediate needs for food and health care. Despite the enforces, adherence to safety protocols was more significant in the capital, and COVID-19 took the inner route in São Paulo State [3]. Two patterns of disease dispersion were described by Fortaleza *et al*. [4]: one by contiguity, in which the virus spreads through areas of conurbation, and another hierarchical (long-distance spread through elementary spatial structures, such as highways, to cities with several degrees of connectivity).

São Paulo State is divided into 17 health departments with respect to epidemiological control, each one represented by a major city: Araraquara, Araçatuba, Baixada Santista, Barretos, Bauru, Campinas, Franca, São Paulo Metropolitan Area, Marília, Piracicaba, Presidente Prudente, Registro, Ribeirão Preto, São João da Boa Vista, São José do Rio Preto, Sorocaba and Taubaté. COVID-19 was introduced in different moments and behaved in different ways in each one of these Regional Health Departments (DRS, in Portuguese). We aimed to study COVID-19 advance in all these regions by analysing new confirmed cases per day (after the first case of COVID-19 in Brazil) and calculating the effective reproduction number (*R_t_*) of SARS-CoV-2 over time. Also, daily new cases of Severe Acute Respiratory Illness (SARI) and its *R_t_* number were estimated as an alternative way to follow the temporal evolution of the disease in a country still struggling to increase testing capacity [5].

Since 27 May, São Paulo State adopted a plan of quarantine measures (‘Plano São Paulo’), which can be more restrictive or more flexible, considering the growth rate of COVID-19 cases and deaths, and bed occupancy rates in each DRS. All cities belonging to a DRS are ruled by the same quarantine measures, which were called phases. Phase 1 (red phase) is considered a contamination phase, in which the rates of spread of the disease are high and, the capacity of the health system is close to its limit, with permission only for essential services. Phase 2 (orange phase) is considered an attention phase, with the possibility of some services opening. For commerce, limit to maximum occupancy 20% of the location's capacity, with reduced hours: 4 h in a row on all days of the week or 6 h in a row on 4 days of the week, always adopting standard and sector-specific protocols. Food courts are still banned in this phase. Phase 3 (yellow) is considered a controlled phase, with some flexibilisation. For example, commerce may open with maximum occupancy limited to 40% of total capacity and reduced hours (8 h). Finally, phase 4 (green), a partial opening phase, in which all services are allowed to open, respecting the limit of 60% of capacity and maintaining all specific protocols. Bars, restaurants, beauty salons and barbershops will only be open from phases 3 and 4, yellow and green.

## Methods

We monitored the number of SARI and confirmed cases of COVID-19, over time, for each Regional Health Department of São Paulo State, Brazil [6]. These permitted us to calculate the effective reproduction number (*R_t_*) for COVID-19 in each of these regions and evaluate the evolution of the epidemic using the methodology proposed by Wallinga and Lipsitch [7]. Unfortunately, data from Brazil does not distinguish imported cases from local cases, making it inviable to use more recent methodologies to estimate *R_t_* [8]. Since 20 March, community transmission of COVID-19 was declared for the whole country, impacting notifications.

Data were obtained from the national database SIVEP-Gripe, which registers all severe hospitalised cases of SARI and identifies confirmed COVID-19 cases, and covers the period from the date of the first confirmed case of COVID-19 (25 February) until 200 days after. A nowcasting procedure [9] was performed to correct delay in notifications that span 40 days before the last case. The last week was ignored in the analysis. After that, the data were smoothed using a moving average with a window of 7 days.

## Results

**Fig. 1.**
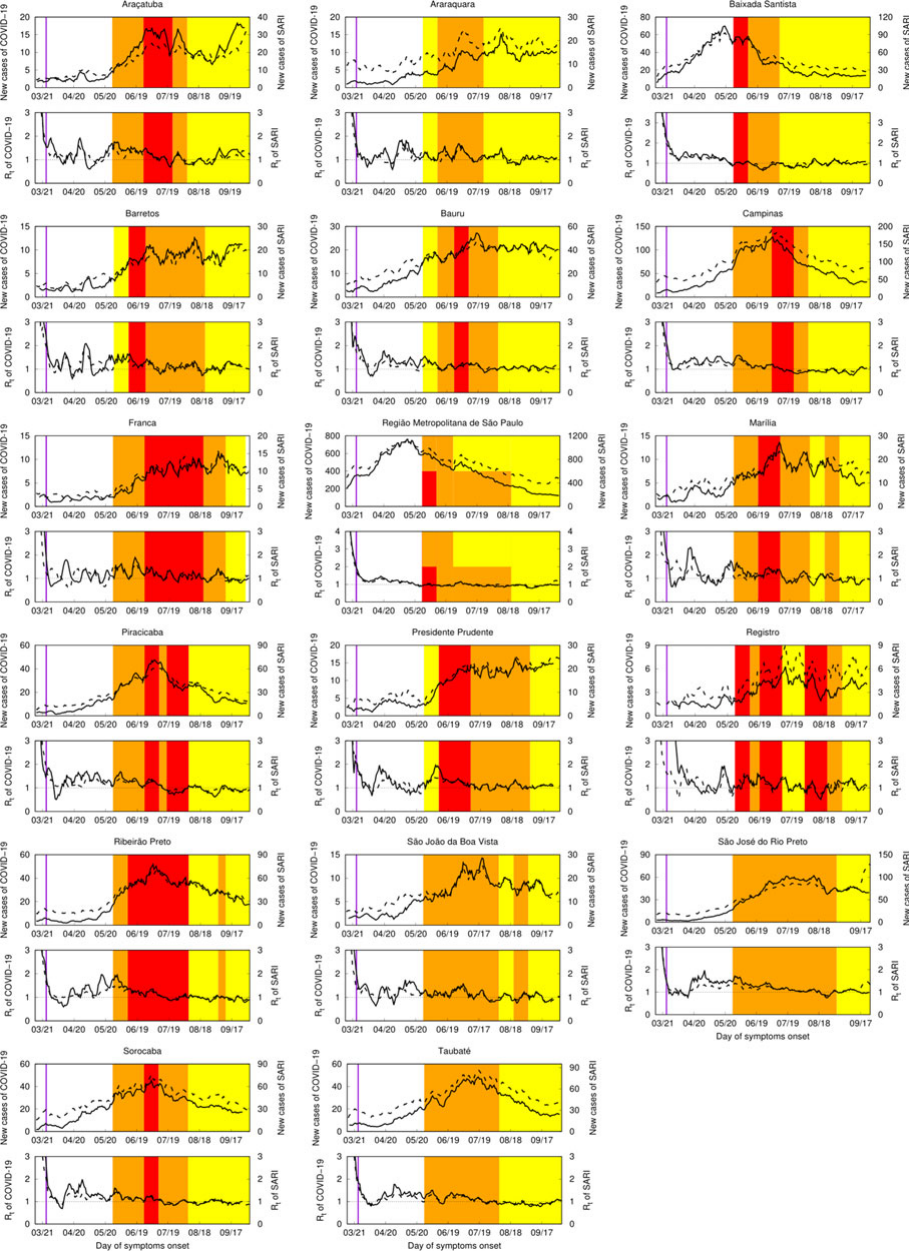
Epidemic evolution of COVID-19 in the Regional Health Departments (DRS) of São Paulo State, Brazil. In each panel identified with the DRS name, top curves correspond to new confirmed cases of COVID-19 in full lines and new notifications for severe acute respiratory illness (SARI) in dashed lines. Bottom curves correspond to the temporal evolution of Rt for COVID-19 (full lines) and SARI (dashed lines). Vertical purple lines mark March 24th, the day of the first quarantine recommendations for São Paulo State. Red, orange, or yellow painted periods represent phases of the São Paulo plan. Phase one is addressed in red, phase 2 in orange, and phase 3 in yellow. None of DRS achieved phase 4 (green), a reopening phase, during the period of this study.

Figure 1 shows the time evolution of new cases, and *R_t_* for both COVID-19 confirmed cases and SARI. The first case of COVID-19 was reported in the DRS of São Paulo Metropolitan Area on 25 February. For SARI, we reported the cases beginning on 15 March, when a change of protocol was done to englobe COVID-19 on SARI notifications. The results obtained for COVID-19 are shown in full lines and for SARI in dashed lines. Vertical lines have been marked to signal the dates for changes in social distancing protocols and trade functioning. The 24 March (purple vertical line) represents the generalised quarantine for the state as a whole. From 27 May on, the São Paulo plan was implemented. Coloured lines indicate the phase in which each DRS belongs. Phases 1, 2 and 3 are represented by the colours red, orange and yellow. None of the DRS was in the reopening phase (phase 4, green) at the end of this study.

One can notice the difference of scales for COVID-19 confirmed cases and SARI notifications to follow the disease's spatial–temporal dynamics in each location. The confirmed cases of COVID-19 struggle with the country's capacity to acquire quick tests, additionally to the shortages of molecular testing supplies. SARI cases can vary from twice as high as COVID-19 cases (e.g. Franca and Registro) to four times higher (e.g. Araquara and Barretos). Grotto *et al.* [5] showed that molecular diagnosis increased in São Paulo State over the epidemiological weeks, but it does not match the much higher increase in the number of cases, challenging diagnostic capacity and, therefore, accurate and timely health surveillance.

The trends in increasing or decreasing epidemic velocity are captured by both measures SARI and COVID-19 (see *R_t_* curves). Oscillations can be clearly observed on the COVID-19 curve of new cases.

## Discussion

Our results confirm the spatial–temporal dispersion of COVID-19 over São Paulo State described by Fortaleza *et al*. [3, 4]. Higher numbers of confirmed COVID-19 cases are seen earlier in São Paulo Metropolitan Area DRS, accompanied by conurbation areas, such as Campinas DRS and Baixada Santista DRS. On 27 May, they belonged to the red phase, indicating high rates of disease spreading and high hospital bed occupancy rates. This is evidence of the earlier introduction of SARS-CoV-2 in these regions and may explicit the contiguity model of disease dispersion.

It is interesting to highlight that the social distance measures and improvement in testing, mask-wearing and standard health security protocols, were adopted and impacted slowing down epidemic velocity in all DRS, but in different moments. In São Paulo Metropolitan Area, protocol adherence was greater in March and April, as we can see the *R_t_*'s reduction over time. In the countryside, rules were not strictly followed in this very first moment because the number of cases and deaths were still not so alarming, giving the population a false security sensation. São Paulo State general quarantine started on 24 March and was extended until 27 May, when the São Paulo plan started. Over this period, all DRS showed *R_t_* values close to 1, but only sometimes below 1. This was not sufficient to stop the epidemic, as the number of confirmed cases kept growing, but was able to slow down dissemination.

When taking special attention to more recent *R_t_* values (June, for instance), we can see the interior of São Paulo State is at a critical phase of the epidemic. Inner regions such as Marilia, Araraquara and Barretos DRS show *R_t_* values sometimes much higher than 1. Bauru shows similar behaviour. An increase in the *R_t_* in these locations is mostly due to more recent disease introduction, not following social distancing measures, and local issues on testing and isolating positive cases. This scenario is not compatible with a plan of reopening commerce and industry activities, which have been induced by some mayors who insist on questioning the São Paulo State quarantine plans.

Finally, we observed that we can follow COVID-19 epidemic behaviour by following SARI notifications. Obviously, there is a great difference on the scale of numbers. As commented before, this might be secondary to a lack of diagnostic power and a delay in the diagnosis of confirmed cases. Anyhow, as we can see in Figure 1, in all DRS, full lines and dashed lines run together and represent remarkably similar curves. A low-to-medium income country with serious structural issues, such as Brazil, looking at SARI incidence and how it increased in different areas might represent an alternative way to estimate the real epidemic's numbers.

Similar studies estimated *R_t_* considering imported and local transmission cases because the first one seems to be particularly important for a newly introduced disease [10, 11]. Such methodology cannot be applied to Brazil's data since this information is not captured in the SARI mandatory notification form. However, the obtained *R_t_* values at the different phases of the epidemic are in the range observed in other studies [10, 11]. Also, as in other countries, slowing down on epidemic spreading was observed when the non-pharmacological measures were introduced, but it was not enough to control the epidemic, differently from many countries. Local issues on epidemic control are still a challenge for Brazilian studies. Delays in notifications, poorly computerised systems, difficulties in contact tracing, open-source data and political and economic fragilities are some. Finally, lock-down was never performed in any location of São Paulo state.

Studying *R_t_* values and relating to the number of confirmed new cases and SARI cases permitted us to evaluate quarantine plans and their impact on disease spreading over time. Along with universal mask-wearing and testing and isolating positive cases, social distance measures were able to diminish epidemic velocity, impacting the reduction of *R_t_*. Still, they were insufficient to stop transmission, as the *R_t_* was mostly established over one, and the number of cases kept growing. Today, after 200 days since the first confirmed case of COVID-19 in Brazil, the São Paulo State situation is still alarming. Although many regions started showing a reduction in the number of new cases since August, *R_t_* on most of these locations is greater than 1, making it clear new strategies on public health and epidemic control urges.

## Data Availability

The authors state that the database used in the analyses can be available as a supplementary file to the paper or provided to interested researchers upon reasonable request.
